# Aberrant Bone Density in Aging Mice Lacking the Adenosine Transporter ENT1

**DOI:** 10.1371/journal.pone.0088818

**Published:** 2014-02-19

**Authors:** David J. Hinton, Meghan E. McGee-Lawrence, Moonnoh R. Lee, Hoi K. Kwong, Jennifer J. Westendorf, Doo-Sup Choi

**Affiliations:** 1 Department of Molecular Pharmacology and Experimental Therapeutics, Mayo Clinic College of Medicine, Rochester, Minnesota, United States of America; 2 Neurobiology of Disease Program, Mayo Clinic College of Medicine, Rochester, Minnesota, United States of America; 3 Department of Psychiatry and Psychology, Mayo Clinic College of Medicine, Rochester, Minnesota, United States of America; 4 Department of Orthopedic Surgery and Orthopedic Research, Mayo Clinic College of Medicine, Rochester, Minnesota, United States of America; 5 Advanced Genomics Technology Center, Mayo Clinic College of Medicine, Rochester, Minnesota, United States of America; University of Colorado Denver, United States of America

## Abstract

Adenosine is known to regulate bone production and resorption in humans and mice. Type 1 equilibrative nucleoside transporter (ENT1) is responsible for the majority of adenosine transport across the plasma membrane and is ubiquitously expressed in both humans and mice. However, the contribution of ENT1-mediated adenosine levels has not been studied in bone remodeling. With the recent identification of the importance of adenosine signaling in bone homeostasis, it is essential to understand the role of ENT1 to develop novel therapeutic compounds for bone disorders. Here we examined the effect of ENT1 deletion on bone density using X-ray, dual energy X-ray absorptiometry and micro-computerized tomography analysis. Our results show that bone density and bone mineral density is reduced in the lower thoracic and lumbar spine as well as the femur of old ENT1 null mice (>7 months) compared to wild-type littermates. Furthermore, we found increased mRNA expression of tartrate-resistant acid phosphatase (TRAP), an osteoclast marker, in isolated long bones from 10 month old ENT1 null mice compared to wild-type mice. In addition, aged ENT1 null mice displayed severe deficit in motor coordination and locomotor activity, which might be attributed to dysregulated bone density. Overall, our study suggests that ENT1-regulated adenosine signaling plays an essential role in lumbar spine and femur bone density.

## Introduction

Bone disorders including senile osteopenia, osteoporosis and diffuse idiopathic skeletal hyperostosis (DISH) are serious public health concerns of the aging population. It is estimated that the cost of osteoporosis and related fractures is about $20 billion annually in the US [1]. Perhaps more striking is that osteoporosis affects an estimated 44 million Americans or 55% of people older than 50 years old [2]. Additionally, the 5-year survival rate is as low as 28% for patients who suffered a vertebral fracture related to osteoporosis [3]. Despite the availability of current pharmaceutical agents for the treatment of senile osteoporosis and the absence of treatment for DISH, the development of new drugs will have a significant impact on patient health [2].

Recently, adenosine signaling has been shown to regulate bone remodeling [4], [5], [6], [7], [8], [9], [10], [11]. Adenosine exerts its function through four G-protein coupled receptors including A1, A2A, A2B and A3. Adenosine receptors are expressed in both osteoclasts and osteoblasts [6], [11]. Based on cultured cell experiments, it has been determined that adenosine A1 receptor activation results in osteoclast differentiation [8], [9], [10], [12]. Furthermore, A2A receptor activation inhibits osteoclast differentiation [11] and is important for osteoblast maturation [7]. Moreover, A2B receptor activation has been shown to promote osteoblast differentiation [4] and its activation and overexpression induced the expression of osteoblast-related genes [7]. In vivo rodent studies have also provided evidence for the involvement of adenosine signaling in bone remodeling. Mice lacking adenosine A1 receptor have increased bone density while mice lacking A2A receptor have reduced bone density [8], [10], [11]. Extracellular adenosine levels themselves are also important in bone remodeling. One way adenosine arrives in the extracellular space is through the breakdown of adenosine 5′-triphosphate (ATP) by ecto-5′-nucleotadase (CD73). Mice lacking CD73 display reduced bone density compared to wild-type littermates [13]. Importantly, adenosine is also transported across cellular membranes by nucleoside transporters. In both humans and mice type 1 equilibrative nucleoside transporter (ENT1) is ubiquitously expressed and is responsible for the majority of adenosine transport [14]. In cell culture experiments, the inhibition or knockdown of ENT1 results in reduced adenosine uptake [15], and in mice lacking ENT1, adenosine uptake is also significantly reduced [16]. Interestingly, mice lacking ENT1 have increased circulating adenosine in the plasma [17]. Furthermore, the loss of ENT1 leads to progressive ectopic mineralization of the upper thoracic and cervical spinal cord resembling diffuse idiopathic skeletal hyperostosis (DISH) in humans [18], however, the effect of ENT1 deletion on bone density in the lower portion of the spinal column (lower thoracic and lumbar vertebrae) and femur remained unknown. In this study, we examined the effect of ENT1 deletion on bone density, motor coordination, locomotor function as well as mRNA expression of genes associated with osteoclast activity.

## Materials and Methods

### Animals

ENT1 null mice were generated as described [19]. We used F2 generation hybrid mice with a C57BL/6J×129X1/SvJ genetic background to minimize the risk of false positives or negatives in behavioral phenotypes that could be influenced by a single genetic background [20]. We used 4–15 month old null and wild-type littermates for experiments. Mice were housed in standard Plexiglas cages with food and water *ad libitum*. The colony room was maintained on a 12 h light/12 h dark cycle with lights on at 6∶00 a.m. Animal care and handling procedures were approved by the Mayo Clinic Institutional Animal Care and Use Committees in accordance with NIH guidelines.

### In vivo X-ray Imaging

X-ray images were obtained on a Mammo DIAGNOSTIC UC Series 54 X-ray Machine (Philips Medical System, Best, Netherlands). Animals were anesthetized with a mixture of ketamine/xylazine (100 and 15 mg/kg, *i.p.*; Sigma, St. Louis, MO) and placed on the platform for X-ray acquisition. X-ray micrographs (MIN-R 200 Film 18×24 cm, Kodak, Rochester, NY) were developed and then digitized using a scanner (Epson Expression 1640 XL, Japan). NIH Image J software (National Institutes of Health, Bethesda, Maryland) was used to quantify the bone density from the X-ray images.

### Micro-computed Tomography

Bone architecture and mineralization were evaluated in L4 lumbar vertebrae of male mice using *ex vivo* micro-computed tomography (microCT) as previously described [21]. Briefly, the central portion of each vertebral body was scanned in 70% ethanol on a µCT35 scanner (Scanco Medical AG, Basserdorf, Switzerland) with 7 µm voxel size using an energy setting of 70 kVp and an integration time of 300 ms. Similarly, the distal femoral metaphysis of each femur was scanned in 70% ethanol at 7 micron resolution, using the same energy settings as for the lumbar vertebrae scans. Scan regions were 233 slices in length (1.63 mm), beginning 1.5 mm proximal to the femoral condyles. The central 100 slices from each scan (located from 2.0 to 2.7 mm proximal to the femoral condyles) were analyzed with the manufacturer’s software to compute trabecular architectural indices. Trabecular bone volume fraction (Tb. BV/TV, %), trabecular number (Tb.N,), trabecular thickness (Tb.Th, mm), and trabecular separation (Tb.Sp, mm) were computed using the manufacturer’s software [22].

### Dual-energy X-ray Absorptiometry (DEXA) Analyses

Bone mineral density (BMD) and percent body fat were assessed longitudinally via dual-energy X-ray absorptiometry (DEXA) scanning (PIXImus, GE Healthcare) of live mice at 5, 6, 8 and 9 months of age as described [21]. Two regions of interest were monitored: lower body (including the lumbar spine, pelvis, and hindquarters) and femoral midshaft.

### Real-time RT-PCR

For mRNA analyses, demarrowed cortical bone segments from femurs and tibias were prepared from 7 and 10 month old mice. Mice were anesthetized with carbon dioxide and long bones were rapidly removed and dissected free of soft tissues. Epiphyses were removed from each mouse and marrow was flushed with saline; cortical bone segments were subsequently flash frozen in liquid nitrogen prior to mRNA isolation. Tissues were homogenized in TRIzol® using a high-speed disperser (Ultra-Turrax T25, IKA). To measure mRNA levels, real-time quantitative RT-PCR was performed with the Bio-Rad MyiQ Single Color Real-Time PCR Detection System (Bio-Rad Laboratories, Hercules, CA) using Bio-Rad iQ SYBR Green Supermix as described [21]. Gene-specific primers for ecto-5′ nucleotadase (Nt5e), adenosine kinase (Adk), adenosine deaminase (Ada), endo-5′ nucleotadase (Gm49), adenosine A2A receptor (Adora2a), adenosine A1 receptor (Adora1), tartrate-resistant acid phosphatase (Acp5, also known as TRAP), runt-related transcription factor 2 (Runx2), type I collagen (Col1a1), osteocalcin (Bglap), and cathepsin K (Ctsk) and GAPDH were designed (primer sequences are available upon request). Relative expression levels for each gene of interest were calculated using the 2^−ΔΔCt^ method [23].

### Accelerated Rotarod

To evaluate the motor function, a standard mouse rotarod treadmill (UGO Basile, Verese, Italy) was used as described with minor modifications [24]. The rotarod was programed to gradually accelerate from 5 rpm to 40 rpm over a 5-min period. Latency to fall from the treadmill was used as a measure of motor incoordination. Each mouse underwent one trial per week over a 5-month period beginning at 7 months of age.

### Open-field Activity Monitoring

Spontaneous locomotor activity was measured in brightly lit (500 lux) Plexiglas chambers (41 cm×41 cm) as described [25], [26], [27]. The chambers were located in sound-attenuating cubicles and equipped with two sets of 16 pulse-modulated infrared photobeams to record X–Y ambulatory movements at a 100 ms resolution (Med Associates, Lafayette, IN). Each mouse was placed at the center of the open-field chamber and recording was started upon mouse movement. Each mouse was given one, one-hour trial per week over a 5-month period beginning at 7 months of age. Mouse body weight was measured using an analytical balance (Denver Instrument, Arvada, CO) with a precision of 0.01 g. Body weight was assessed weekly over the 5 month period beginning at 7 months of age.

### Statistical Analysis

All data were expressed as mean ± standard error of the mean (SEM). For bone density measurements, statistics were done by two-tailed Student’s *t* test. For the rotarod, open-field locomotor activity and dual energy X-ray absorptiometry, statistical analyses were carried out by two-way repeated measures ANOVA followed by Tukey *post-hoc* test. For gene expression, non-parametric Mann-Whitney test was used. Criterion for statistical significance was *P*<0.05.

## Results

### Bone Density is Dysregulated in Aged ENT1 Null Mice

**Figure 1:**
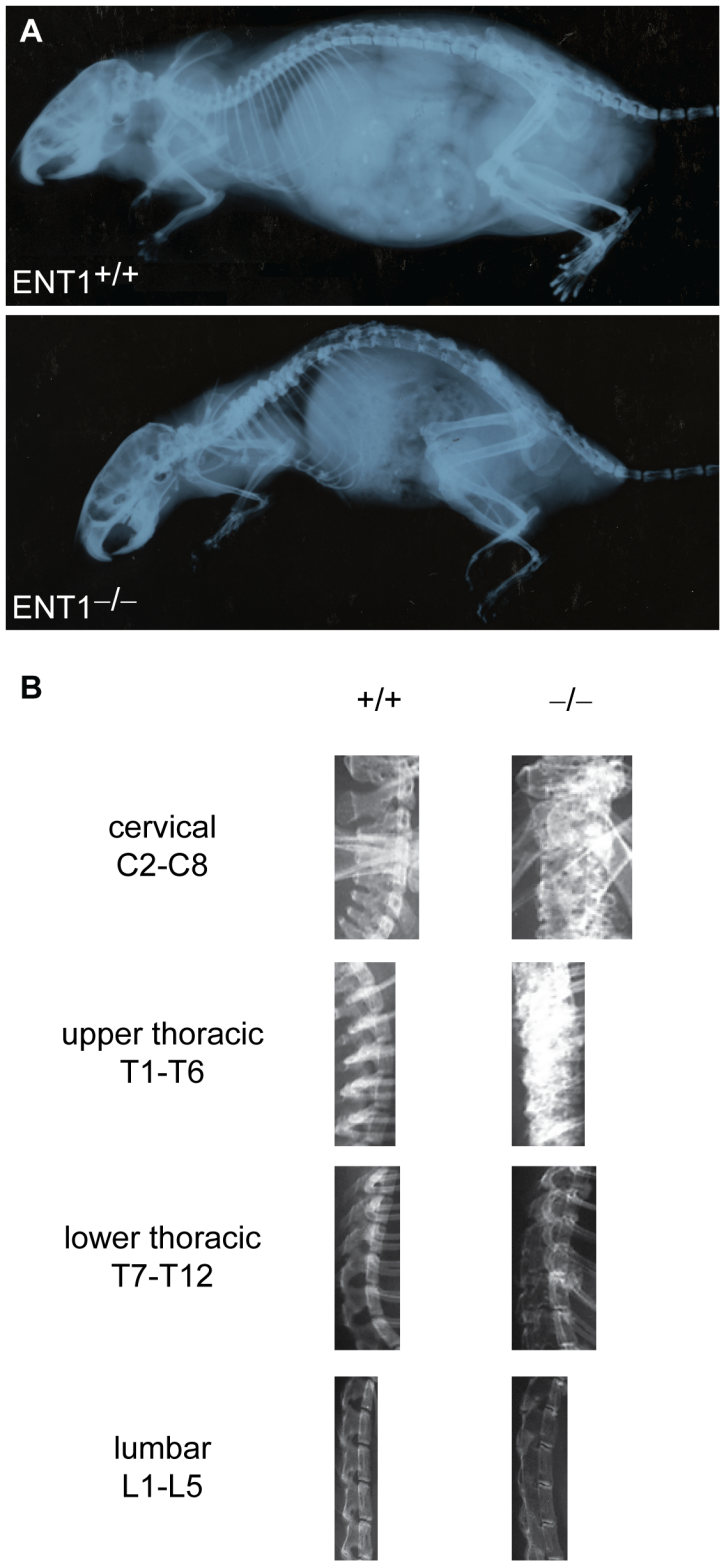
Bone density was assessed using X-ray. **A** ENT1 wild-type and null mice were scanned with X-ray to investigate bone density. Representative X-ray radiographs from an ENT1 wild-type and null mice. **B** For analysis with NIH Image J software, the spinal column was divided into cervical (C2–7), upper thoracic (T1–T6), lower thoracic (T7–T12) and lumbar (L1–L5).

**Figure 2:**
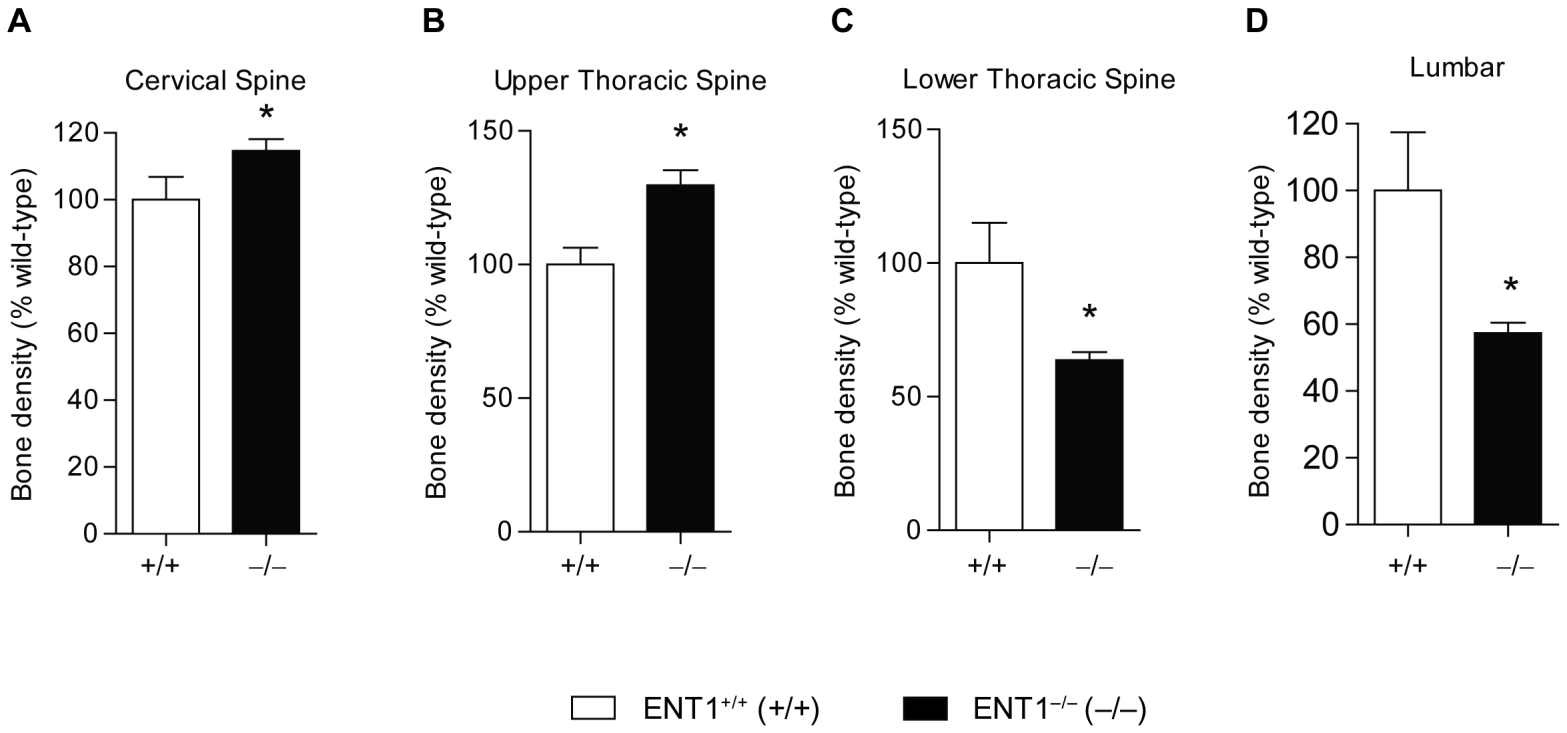
Quantification of bone density in ENT1 null and wild-type mice. **A** Bone density was significantly increased in the cervical spine in ENT1 null (*n* = 12) compared with wild-type (*n* = 5) mice at 7 months of age. **B** Upper thoracic (T1–T6) was significantly increased in ENT1 null mice (*n* = 13) compared to wild-type mice (*n* = 5) at 7 months of age. **C** Bone density in the lower thoracic (T7–T12) was reduced in ENT1 null mice (*n* = 13) compared to wild-type littermates (*n* = 5) at 7 months of age. **D** Lumbar spine bone density was reduced in ENT1 null mice (*n* = 13) compared to wild-type mice (*n* = 5) at 7 months of age. *P<0.05 by two-tailed Student’s *t* test.

First, we measured bone density by X-ray analysis at 7 months of age (Fig. 1A). For quantification, the spine was separated into cervical (C2–7), upper thoracic (T1–6), lower thoracic (T7–12), and lumbar (L1–5) (Fig. 1B) regions, and bone density was estimated via radiographic densitometry. At 7 months of age, there was a significant increase in bone density in the cervical [*t*(15)* = *2.15, *P*<0.05; Fig. 2A] and upper thoracic [*t*(16)* = *2.97, *P*<0.01; Fig. 2B] spinal vertebrae for ENT1 null mice compared to wild-type littermates. On the other hand, the lower spinal column, which includes the lower thoracic [*t*(16)* = *3.59, *P*<0.01; Fig. 2C] and lumbar [*t*(16)* = *3.72, *P*<0.01; Fig. 2D] spine, had reduced bone density in ENT1 null mice compared to wild-type mice.

### Reduced Trabecular Bone Thickness of Lumbar Vertebral Bodies in ENT1 Null Mice

**Figure 3:**
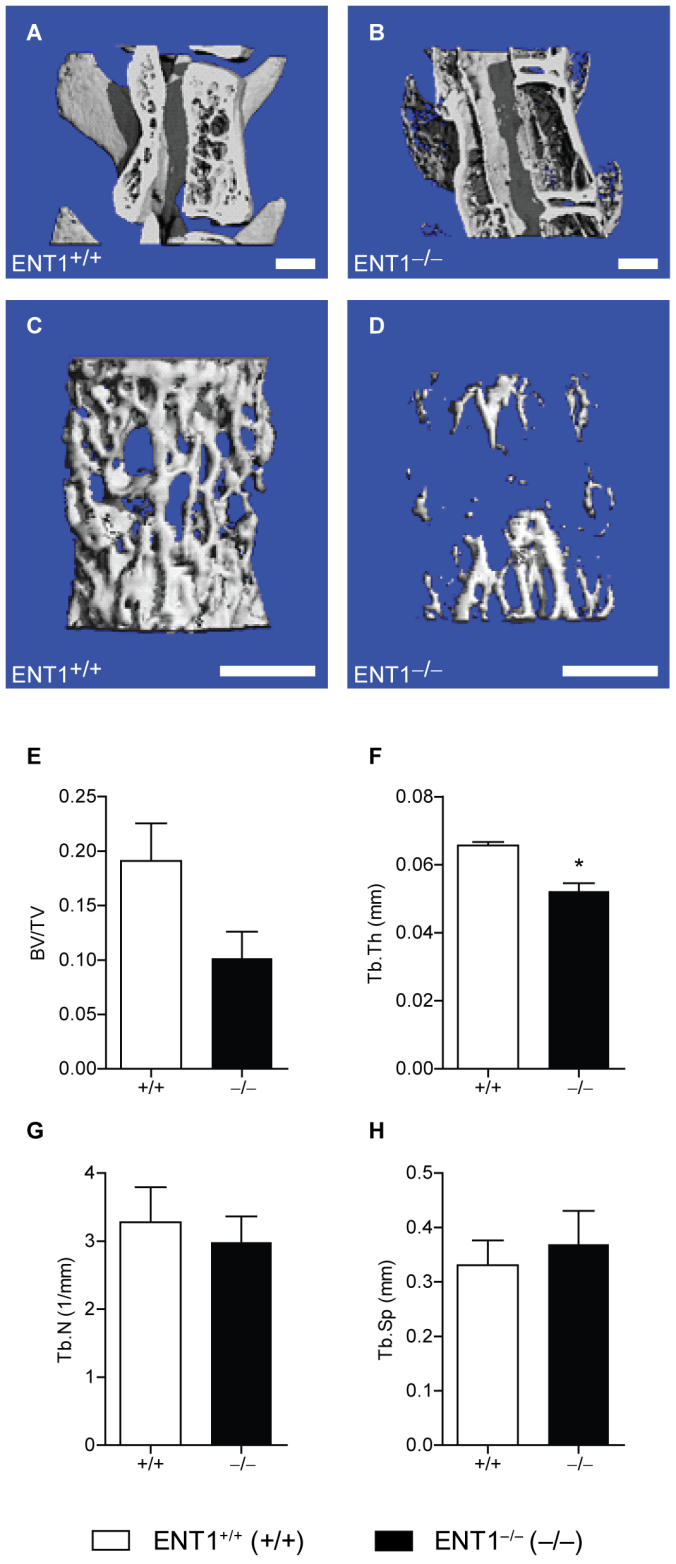
ENT1 null mice present with significantly reduced thickness of trabecular bone within the lumbar vertebral body by micro computerized tomography (microCT). Representative cross section through the vertebral body of a lumbar spine in (**A**) wild-type and (**B**) ENT1 null mice. Scale bar = 1 mm. It was noted that ENT1 null mice have reduced trabecular bone thickness and reduced intervertebral space. Magnified view of the trabecular bone within the lumbar vertebral vertebra of (**C**) wild-type and (**D**) ENT1 null mice. Scale bar = 1 mm. **E** Trend for a reduction in the trabecular bone volume fraction (BV/TV), which is equal to the percentage of the tissue space that is occupied by bone within the lumbar vertebral body, of ENT1 null mice compared to wild-type mice. **F** Trabecular thickness (Tb.Th) or the average thickness of the trabecular struts was reduced in ENT1 null mice compared to wild-type littermates. **G** Trabecular number (Tb.N) or a measure of the number of trabeculae within a given space was similar between wild-type and ENT1 null mice. **H** Trabecular separation (Tb.S) or the average spacing between adjacent trabeculae was similar between ENT1 null and wild-type mice. *n* = 4–5 per genotype. *P<0.05 by Student’s two-tailed *t* test. All data are presented as mean ± SEM.

Because ENT1 null mice had reduced radiographic bone density in the lumbar vertebrae by X-ray analysis, we investigated bone mass and architecture of the lumbar vertebrae in ENT1 null and wild-type mice by micro-computed tomography (microCT). Both cortical and trabecular aspects of the lumbar vertebrae in ENT1 null mice were clearly compromised compared to wild-type littermates (Fig. 3A compared to Fig. 3B). Trabecular bone volume fraction (BV/TV) tended to be lower in ENT1 null mice compared to wild-type mice [*t*(7)* = *2.18, *P = *0.07; Fig. 3C–E] due largely to a significant decrease in trabecular thickness (Tb.Th) [*t*(7)* = *4.77, *P*<0.01; Fig. 3F]. Trabecular number (Tb.N) and trabecular separation, in contrast, showed few differences between groups [Tb.N: *t*(7)* = *0.47, *P = *0.67; Fig. 3G; Tb.Sp: *t*(7)* = *0.48, *P = *0.65; Fig. 3H].

### Reduced Trabecular Bone Volume Fraction, Thickness and Number and Increased Trabecular Separation in the Femur of ENT1 Null Mice

**Figure 4:**
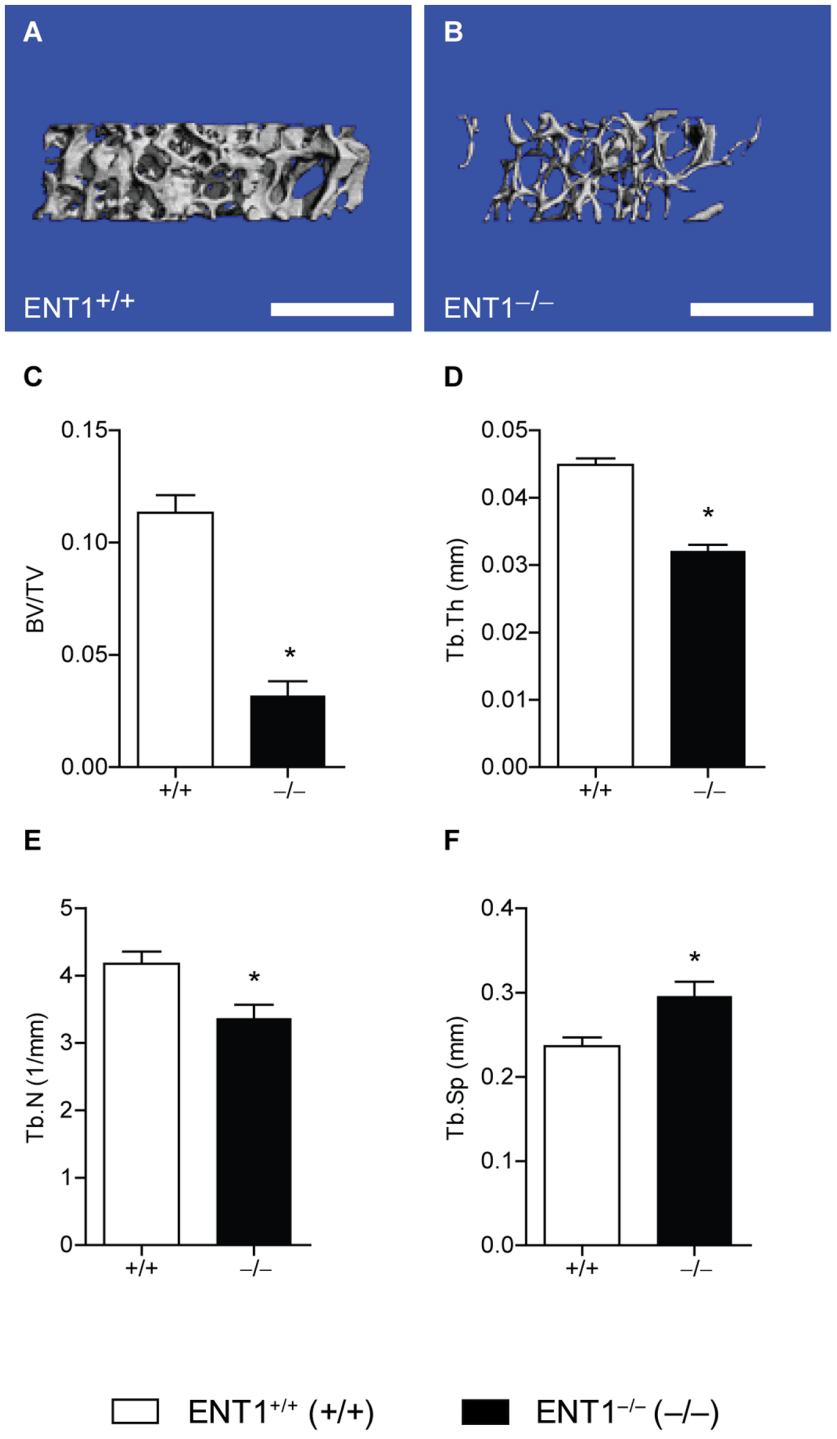
ENT1 null mice have reduced trabecular bone volume fraction, thickness and number and increased trabecular separation in the femur by micro computerized tomography (microCT). Representative cross section through the trabecular bone of the distal femoral metaphysis of the femur of (**A**) wild-type and (**B**) ENT1 null mice. Scale bar = 1 mm. **C** Reduced trabecular bone volume fraction (BV/TV), which is equal to the percentage of the tissue space that is occupied by bone within the lumbar vertebral body, of ENT1 null mice compared to wild-type mice. **D** Trabecular thickness (Tb.Th) or the average thickness of the trabecular struts was reduced in ENT1 null mice compared to wild-type littermates. **E** Trabecular number (Tb.N) or a measure of the number of trabeculae within a given space was decreased in ENT1 null mice relative to wild-type mice. **F** Trabecular separation (Tb.S) or the average spacing between adjacent trabeculae was reduced in ENT1 null compared to wild-type mice. *n* = 3 per genotype. *P<0.05 by Student’s two-tailed *t* test. All data are presented as mean ± SEM.

We next examined bone volume and architecture in the distal femoral metaphysis of the femur of ENT1 null mice. Femoral trabecular bone in ENT1 null mice was clearly compromised relative to that of wild-type mice (Fig. 4A and B). Trabecular bone volume fraction (BV/TV) was decreased in ENT1 null mice compared to wild-type mice [*t*(4)* = *8.15, *P*<0.001; Fig. 4A–C]. This reduction in the trabecular bone fraction appeared to be owing to a significant decrease in both trabecular thickness (Tb.Th) [*t*(4)* = *9.76, *P*<0.001; Fig. 3D] and trabecular number (Tb.N) [*t*(4)* = *3.04, *P = *0.04; Fig. 3E] in ENT1 null mice relative to wild-type mice. Consistently, trabecular separation was increased in ENT1 null compared to wild-type mice [*t*(4)* = *2.97, *P = *0.04; Fig. 4F].

### Reduced Femur Bone Mineral Density in ENT1 Null Mice

**Figure 5:**
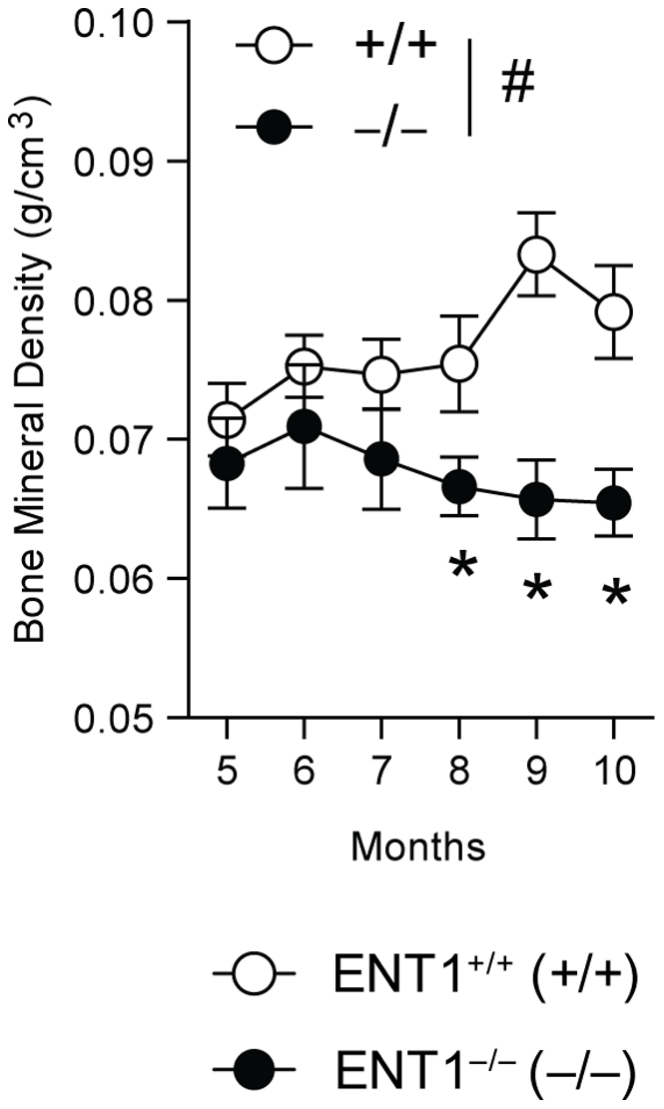
Femur bone mineral density was reduced in ENT1 null mice (*n* = 5–6) compared to wild-type littermates (*n* = 4–6) from 5 to 10 months of age. ^#^ *P*<0.05 for significant main effect of genotype by two-way repeated measures ANOVA. *P<0.05 by two-way repeated measures ANOVA followed by Tukey *post hoc* test for individual comparisons. All data presented as mean ± SEM.

Because the bone architecture was dysregulated in the femur of ENT1 null mice compared to wild-type mice, we investigated temporal changes in ENT1 null mouse bone mass to longitudinally quantify trends in bone mineral density with age using dual energy X-ray absorptiometry (DEXA) in the femoral mid-diaphysis. Bone mineral density (g/cm^3^) was reduced in aged ENT1 null mouse femurs as compared to wild-type mice (Fig. 5) beginning at 8 months of age. Two-way repeated measures ANOVA indicated a significant effect of genotype [F_(1, 39)_ = 14.647, *P*<0.01] and the interaction between genotype and age [F_(5, 39)_ = 1.137, *P* = 0.36] without a significant effect of age [F_(5, 39)_ = 3.361, *P* = 0.01]. Tukey *post-hoc* analysis showed that ENT1 null mice had significantly reduced femur bone mineral density from 8–10 months of age and weighed significantly less, starting when mice reached 7–8 months (Fig. 5).

### Gene Expression Reflects Increased Bone Turnover in the Femur of Aged ENT1 Null Mice

**Figure 6:**
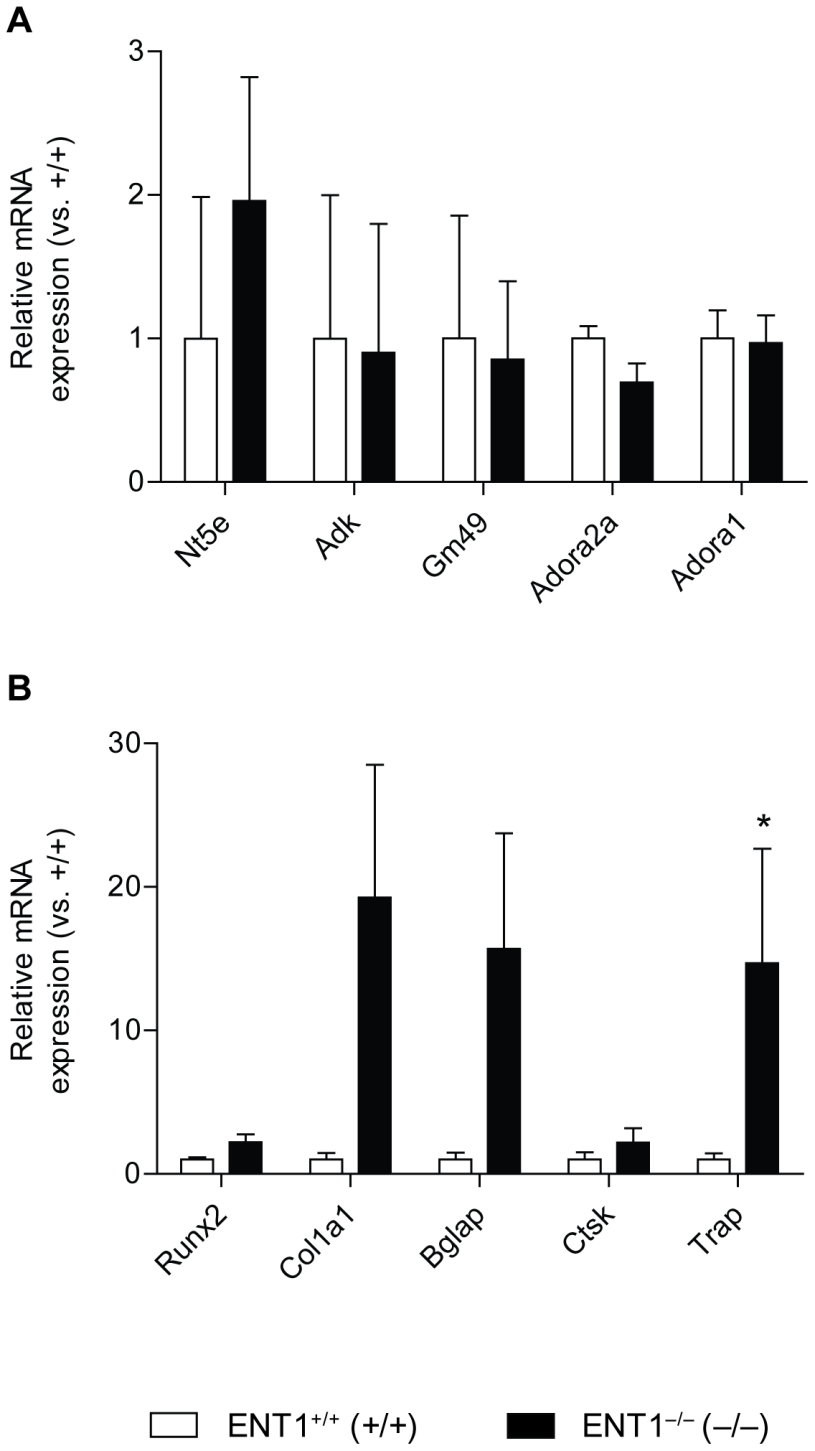
Increased tartrate-resistant acid phosphatase (TRAP) mRNA levels in 1o month old ENT1 null mice. **A** mRNA levels from cortical bone (femur) of genes involved in adenosine signaling. No difference in levels of ecto-5′ nucleotadase (Nt5e), adenosine kinase (Adk), adenosine deaminase (Ada), endo-5′ nucleotadase (Gm49), adenosine A2A receptor (Adora2a) or adenosine A1 receptor (Adora1) mRNA levels when comparing wild-type (*n* = 9) and ENT1 null mice (*n* = 6). **B** Increased tartrate-resistant acid phosphatase (TRAP) mRNA levels in ENT1 null mice compared to wild-type littermates. No difference in levels of runt-related transcription factor 2 (Runx2), alpha-1 type I collagen (Col1a1), osteocalcin (Bglap), or cathepsin K (Ctsk) mRNA levels when comparing wild-type (*n* = 9) and ENT1 null mice (*n* = 6). *P<0.05 by Mann-Whitney test. All data are presented as mean ± SEM.

As bone mineral density was significantly lower in the femoral midshaft of ENT1 null mice, we chose this site to investigate gene expression trends that could help explain the biological mechanisms behind this phenomenon. First, since ENT1 is the main adenosine transporter, we examined whether genes in the adenosine signaling pathway were dysregulated in bone tissue. To examine if ENT1 deficiency alters mRNA expression of adenosine receptors, we chose two representative adenosine receptors, A1 and A2A receptors, which are coupled to G_i_ and G_s_, respectively. Surprisingly, no difference in levels of ecto-5′ nucleotadase (Nt5e), adenosine kinase (Adk), adenosine deaminase (Ada), endo-5′ nucleotadase (Gm49), adenosine A2A receptor (Adora2a) or adenosine A1 receptor (Adora1) mRNA levels when comparing wild-type and ENT1 null mice (Fig. 6A). Next, we investigated genes that are markers of osteoclast and osteoblast behavior. Interestingly, we found significantly increased tartrate-resistant acid phosphatase (TRAP) mRNA levels in ENT1 null mice compared to wild-type littermates (*P* = 0.04; Fig. 6B), suggesting an increase in osteoclastic bone resorption. In addition, a trend was found for increased expression of type I collagen (Col1a1), the primary organic matrix component of bone, in ENT1 null mice compared to wild-type mice (*P* = 0.06; Fig. 6B), suggesting high bone formation activity as well in the femur of ENT1 null animals. Taken together, these data point to a potential mechanism of high bone turnover in ENT1 null mice, which could explain the bone phenotype observed via radiography, DEXA, and microCT No significant differences were seen in levels of runt-related transcription factor 2 (Runx2), osteocalcin (Bglap), or cathepsin K (Ctsk) mRNA levels when comparing wild-type and ENT1 null mice (Fig. 6B), although ENT1 null mice tended to have higher expression of each gene.

### Aged Mice Lacking ENT1 have a Lower Body Weight

**Figure 7:**
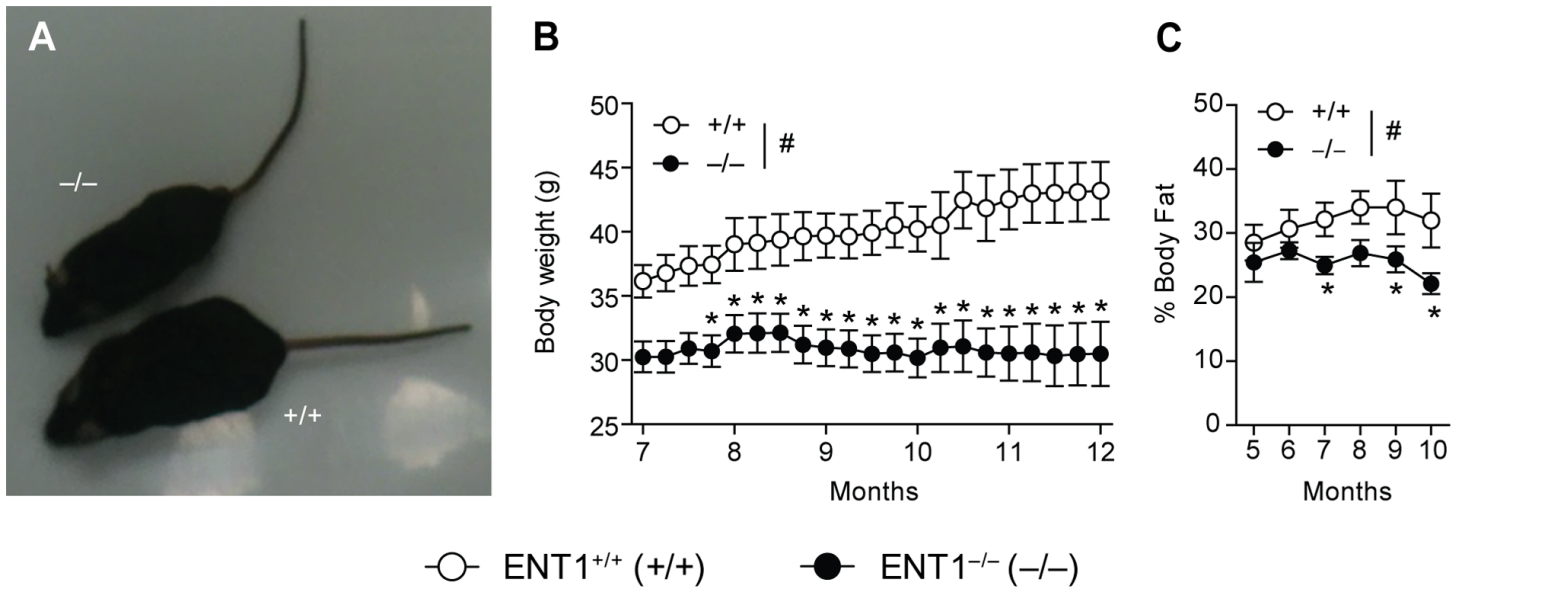
Reduced body weight in aged ENT1 null mice. **A** ENT1 null mice have a smaller body size compared to wild-type littermates at 9 months of age. **B** When body weight was measured weekly, it became apparent that ENT1 null mice (*n* = 16) had significantly reduced body weigh compared to wild-type littermates (*n* = 15) from 7 months 3 weeks of age. **C** ENT1 null mice (*n* = 4–6) have reduced percent body fat compared to wild-type mice (*n* = 4–6). ^#^ *P*<0.05 for significant main effect of genotype by two-way repeated measures ANOVA. *P<0.05 by two-way repeated measures ANOVA followed by Tukey *post hoc* test for individual comparisons. All data presented as mean ± SEM.

We also observed that ENT1 null mice are smaller than wild-type littermates as they aged (Fig. 7A). The body weight of ENT1 null mice is significantly reduced compared to wild-type littermates (Fig. 7B). Two-way repeated measures ANOVA indicated a significant effect of genotype [F_(1, 476)_ = 17.277, *P*<0.001], age [F_(20, 476)_ = 2.695, *P*<0.001] and the interaction between genotype and age [F_(20, 476)_ = 5.562, *P*<0.001]. Tukey *post-hoc* analysis revealed that ENT1 null mice weighed significantly less, especially when mice reached 7–8 months (Fig. 7B). Furthermore, we quantified percent body fat from our DEXA experiment. We found that ENT1 null mice had significantly lower percent body fat compared to wild-type littermates (Fig. 7C) at 7, 9 and 10 months of age. Two-way repeated measures ANOVA showed a significant effect of genotype [F_(1, 39)_ = 8.196, *P* = 0.02], age [F_(5, 39)_ = 3.101, *P* = 0.02] and the interaction between genotype and age [F_(5, 39)_ = 2.666, *P* = 0.04].

### Reduced Accelerating Rotarod Performance but Normal Locomotor Activity in Aged ENT1 Null Mice

**Figure 8:**
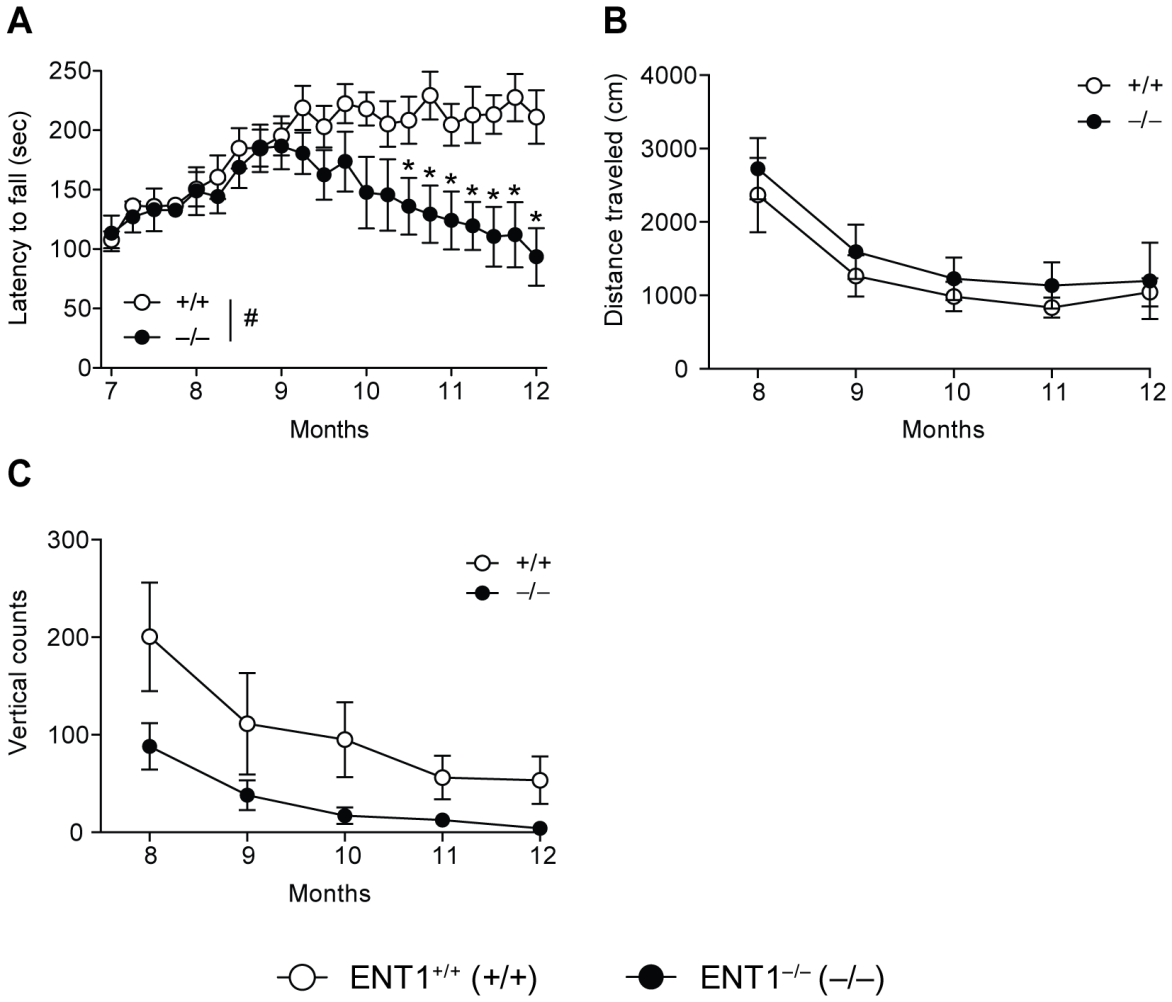
Reduced motor coordination but normal locomotor behavior in ENT1 null mice. **A** ENT1 null mice (*n* = 16) began to fall off the accelerating rotatod sooner than wild-type mice (*n* = 15) by 10.5 months. There is no difference in open-field locomotor activity (**B**) or vertical counts in an open field chamber (**C**) between ENT1 null (*n* = 9) and wild-type mice (*n* = 10). *P<0.05 by two-way repeated measures ANOVA followed by Tukey *post hoc* test for individual comparisons. All data presented as mean ± SEM.

Since bone density was differentially altered in ENT1 null mice compared to wild-type littermates, we investigated the consequence of this dysregulated bone density on motor incoordination. As ENT1 null mice age (>10 months), they are more likely to be unable to remain on an accelerating rotarod bar compared to wild-type littermates (Fig. 8A). Two-way repeated measures ANOVA showed significant effects of genotype [F_(1,402)_ = 5.016, *P* = 0.03], age [F_(20,402)_ = 4.553, *P*<0.001] and the interaction between genotype and age [F_(20,402)_ = 3.980, *P*<0.001]. Tukey *post-hoc* analysis indicated that ENT1 null mice fell off the rotating bar more frequently beginning at approximately 10 months of age. Open-field activity boxes were used to measure distance traveled and vertical counts. There was no difference between ENT1 null and wild-type littermates in the distance traveled (cm) as mice aged (Fig. 8B). Two-way repeated measures ANOVA revealed a significant effect of age [F_(4, 58)_ = 18.907, *P*<0.001] but did not show a significant effect of genotype [F_(1, 58)_ = 0.059, *P* = 0.81] or the interaction between genotype and age [F_(4, 58)_ = 0.728, *P* = 0.58]. This suggests that overall, both wild-type and ENT1 null mice displayed age-dependent reduction of locomotion in the open field-chambers over time. In addition, rearing behavior was measured by determining the number of vertical counts of each mouse. There was not a statistically significant difference in the number of vertical counts between ENT1 null and wild-type mice. However, there was a trend for reduced vertical counts in ENT1 null mice compared to wild-type littermates (Fig. 8C). Two-way repeated measures ANOVA showed a significant effect of age [F_(4, 62)_ = 15.579, *P*<0.001] and a trend for a significant effect of genotype [F_(1, 62)_ = 3.896, *P* = 0.07], but did not show a significant interaction between genotype and age [F_(4, 62)_ = 1.013, *P* = 0.41]. Consistent with locomotor activity (Fig. 8B), mice show aging-dependent decrease of the rearing behavior in both genotypes.

## Discussion

In present study, we focused on the lower portion of the spine and femur. Mice lacking ENT1 exhibit reduced bone density in the lower half of the spinal column as well as in the midshaft of the femur. We also confirmed that ENT1 null mice also presented with increased bone density in the cervical and upper thoracic vertebrae, which is consistent the previous finding by Warraich *et al.* illustrating the presence of osteoid formations in the upper portion of the spinal column of ENT1 null mice [18]. Furthermore, ENT1 null mice weighed significantly less and had reduced percent body fat than wild-type mice.

Interestingly, 2–4 month old ENT1 null mice do not show notable behavioral deficits as they exhibit similar baseline performance in a battery of commonly used behavioral assays to measure motor coordination (rotarod) and general locomotor activity (open-field chambers) [25], suggesting a potential age-dependent ENT1 function that may contribute to decreased bone density. Since a line of previous findings illuminate the importance of adenosine levels in bone formation and resorption, regulation of ENT1 function or expression might be highly relevant as a novel treatment target for bone diseases. It remains to be determined whether the observed bone phenotype results in a reduction in rotarod performance or if the deficit in movement in ENT1 null mice results in a reduction in bone density. It is noteworthy that, based on our DEXA experiments, we observed a reduction in bone mineral density by 8 months of age which precedes the presentation of a behavioral deficit which occurred at around 10.5 months of age.

It is important that ENT1 null mice have significantly lower body mass and reduced percent body fat (Fig. 7). It may be possible that the reduced body weight of ENT1 null mice contributes, in part, the observed reduction of bone density. It is likely that smaller animals will have less bone, which would be reflected in the BMD measurements from our DEXA experiment, especially in the midshaft of the femur (which is an area based measurement). However, the microCT measurements are 3-dimensional, and thus we measured bone density normalized to tissue volume, and therefore corrected for size differences.

Adenosine signaling appears to control the differentiation and maturation of both osteoblasts and osteoclasts [4], [5], [6], [7], [8], [9], [10], [11]. However, despite the biological significance of adenosine signaling in bone remodeling, the origin of extracellular adenosine in bone tissue has remained controversial. Previous studies have examined the importance of the metabolism of adenosine 5′-triphosphate (ATP) to adenosine by ecto-5′-nucleotadase (CD73) as an important regulator of extracellular adenosine levels, which contribute to bone remodeling. This is evident, as mice lacking CD73 have slightly reduced bone density compared to wild-type littermates [13]. However, adenosine is also readily transported directly across cellular membranes through ENTs. Since ENT1 is responsible for the majority of adenosine transport and is ubiquitously expressed [14], our present finding highlights the importance of ENT1 in bone homeostasis as these mice have reduced bone density and bone mineral density in the lower spine and femur.

We also found that markers of osteoclast activity are increased in femur samples isolated from ENT1 null mice. Bone formation activity, reflected by expression of type 1 collagen, also tended to be higher in ENT1 null mice, suggesting that remodeling activity remained coupled in ENT1 null mice. However, in light of the reduced bone mineral density seen in the lumbar vertebrae and femur in ENT1 null mice compared to wild-type littermates, these data point to a potential mechanism of high bone turnover in ENT1 null animals, whereby bone resorption is disproportionately higher than formation which subsequently reduces bone mass. The result of high-turnover osteoporosis is also consistent with our microCT data using lumbar vertebrae that illustrated that trabecular thickness (Tb.Th) was lower in the ENT1 null mice compared to wild-type mice.

Alcoholism and osteoporosis or dysregulated bone density are commonly co-occurring diseases and excessive ethanol administration in rodents has even been used to model osteoporosis [28], [29]. It is noteworthy that mice lacking ENT1 are genetically predisposed to drink more ethanol compared to wild-type littermates [19], [30], [31] and also appear to develop abnormal bone density, even in the absence of ethanol exposure as with the present study. It may be possible that adenosine signaling could be a link between ethanol consumption and altered bone density. Acute ethanol exposure is known to block adenosine uptake in neurons [32] and peripheral tissues [33] resulting in increased extracellular adenosine levels. Furthermore, adenosine receptor agonist potentiates ethanol-induced ataxia indicating that adenosine signaling mediates the ataxic effect of ethanol (reviewed in [34]). However, chronic ethanol exposure no longer inhibits adenosine transporting activity with decrease of ENT1 expression and function [35], indicating that adenosine levels are differentially regulated depending on the nature of exposure or ethanol doses. Interestingly, chronic, but moderate alcohol consumption is associated with increased bone density while chronic-excessive alcohol consumption has been shown to result in reduced bone density (Reviewed in [36]). Rodent studies have highlighted the relationship between chronic ethanol consumption and reduced bone density since chronic ethanol reduces bone growth, bone development, bone formation and the mechanical properties of bones in rats [37], [38], [39].

Notably aberrant bone density was only observed in older ENT1 null mice, but not in younger ENT1 null mice. Since the most bone-related disorders including osteoporosis, osteopenia or DISH are growing problems of the world’s elderly population [2], [40], it is interesting to note the differential, but controversial age-related changes in adenosine receptor expression. Castillo and colleges reported an age-related loss of adenosine A1 receptors while A2b receptors were increased in senescence-accelerated mice (SAM) resistant strain 1 (SAMR1) [41]. In addition, in a mouse model of accelerated aging [SAM prone strain (SAMP8)], levels of A1 receptor are reduced compared to the SAMR1 mice at a young age (3 weeks) [41]. Consistently, human studies have shown that there is an age-related decline in adenosine A1 receptor, but not A2A, in the striatum [42]. However, as reviewed by Cunha, it appears that A2A receptor may be increased in some areas of the basal ganglia in aged rats [43]. In addition, extracellular adenosine levels appear to be reduced in aged animals suggesting that there may be changes in the transport of adenosine or adenosine metabolism with the aging process [43]. Interestingly, SAMP mice have been used as a model of osteoporosis [44], [45], [46], [47], [48], [49], [50]. Importantly, osteoclasts appear to be increased in SAMP6 mice, which may explain the decline in bone density [48]. Since A1 receptor activation has been shown to lead to osteoclast differentiation [9], [10], [12], the age related decline in A1 receptors observed in SAMR1 mice and the initial difference between SAMR1 and SAMP8 mice as well as the increase in A2B receptor expression with age [41] might be a compensatory mechanism to maintain bone homeostasis.

Adipocytes differentiate from mesenchymal stem cells (MSCs) in an adenosine receptor dependent manner. Adenosine A1 and A2A receptor activation are associated with increased adipogenesis from rat bone marrow mesenchymal stem cells [7]. Furthermore, expression of A1 receptors induced adipocyte differentiation while A2B receptor expression inhibited adipogenesis in 7F2 cells transfected with these receptors [51]. Therefore, if ENT1 null mice had reduced A2A receptor activity or adenosine levels insufficient to activate A2A receptor they would potentially have reduced adipogenesis as well as less inhibition of osteoclast differentiation. Additional research is required to confirm the role of the adenosine A2A receptor in bone density and body weight.

In summary, our findings suggest that deficiency of ENT1 contributes to reduced bone density in aged mice, which appears to be, in part, associated with deficits in motor coordination and dysfunctional locomotor activity in ENT1 null mice.
